# *cis*-Tri­aqua­[1,1′-(propane-1,3-di­yl)bis­(pyridin-1-ium-4-carboxyl­ato)-κ*O*]bis­(thio­cyanato-κ*N*)manganese(II) dihydrate

**DOI:** 10.1107/S1600536814000701

**Published:** 2014-01-18

**Authors:** Qing-Hua Tan, Yan-Qin Wang

**Affiliations:** a College of Chemistry and Chemical Engineering, Department of Chemistry, Inner Mongolia University, Huhhot 010021, People’s Republic of China

## Abstract

In the title compound, [Mn(NCS)_2_(C_15_H_14_N_2_O_4_)(H_2_O)_3_]·2H_2_O, the metal ion is octa­hedrally coordinated by three water mol­ecules, one carboxyl­ate O atom from a 1,1′-(propane-1,3-di­yl)bis­(pyridinium-4-carboxyl­ate) ligand and two N atoms from two thio­cyanate anions in *cis* positions, forming a mononuclear complex mol­ecule. In the crystal, mol­ecules are connected into a three-dimensional architecture through O—H⋯O hydrogen bonds involving water mol­ecules and carboxyl­ate groups.

## Related literature   

For related literature concerning the ligand, see: Jiang & Li (2006 ▶); Li *et al.* (2007 ▶); Wu *et al.* (2006 ▶); Zhang *et al.* (2002 ▶).
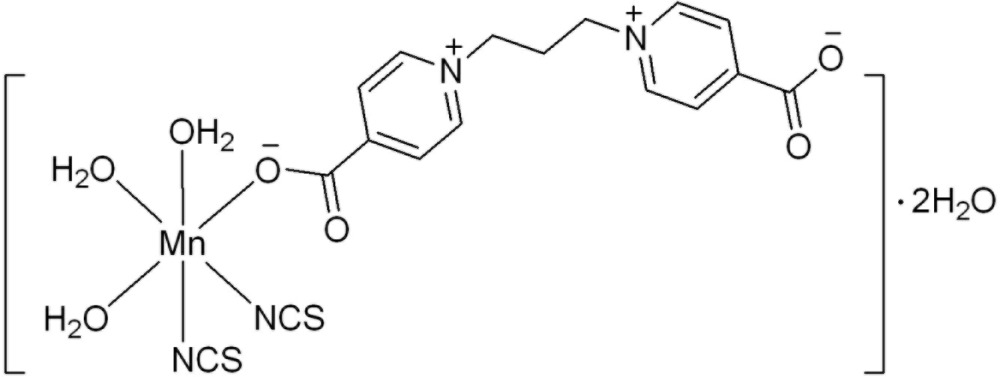


## Experimental   

### 

#### Crystal data  

[Mn(NCS)_2_(C_15_H_14_N_2_O_4_)(H_2_O)_3_]·2H_2_O
*M* *_r_* = 547.46
Monoclinic, 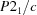

*a* = 17.056 (2) Å
*b* = 11.7514 (16) Å
*c* = 11.8962 (16) Å
β = 92.984 (2)°
*V* = 2381.1 (6) Å^3^
*Z* = 4
Mo *K*α radiation
μ = 0.78 mm^−1^
*T* = 273 K
0.10 × 0.08 × 0.05 mm

#### Data collection  

Bruker SMART CCD area-detector diffractometer
Absorption correction: multi-scan (*SADABS*; Bruker, 1999 ▶) *T*_min_ = 0.926, *T*_max_ = 0.962
14572 measured reflections
5451 independent reflections
3409 reflections with *I* > 2σ(*I*)
*R*_int_ = 0.049

#### Refinement  

*R*[*F* ^2^ > 2σ(*F* ^2^)] = 0.045
*wR*(*F* ^2^) = 0.104
*S* = 1.04
5451 reflections
328 parameters
6 restraints
H atoms treated by a mixture of independent and constrained refinement
Δρ_max_ = 0.34 e Å^−3^
Δρ_min_ = −0.33 e Å^−3^

### 

Data collection: *SMART* (Bruker, 1999 ▶); cell refinement: *SAINT-Plus* (Bruker, 1999 ▶); data reduction: *SAINT-Plus*; program(s) used to solve structure: *SHELXTL* (Sheldrick, 2008 ▶); program(s) used to refine structure: *SHELXTL*; molecular graphics: *SHELXTL*; software used to prepare material for publication: *SHELXTL* and local programs.

## Supplementary Material

Crystal structure: contains datablock(s) I, New_Global_Publ_Block. DOI: 10.1107/S1600536814000701/bt6955sup1.cif

Structure factors: contains datablock(s) I. DOI: 10.1107/S1600536814000701/bt6955Isup2.hkl

CCDC reference:

Additional supporting information:  crystallographic information; 3D view; checkCIF report

## Figures and Tables

**Table 1:** Hydrogen-bond geometry (Å, °)

*D*—H⋯*A*	*D*—H	H⋯*A*	*D*⋯*A*	*D*—H⋯*A*
O5—H5*WA*⋯O3^i^	0.89 (4)	1.84 (4)	2.707 (3)	164 (4)
O5—H5*WB*⋯O3^ii^	0.81 (4)	1.88 (4)	2.682 (3)	174 (4)
O6—H6*WA*⋯O9^iii^	0.78 (3)	2.09 (3)	2.862 (3)	168 (4)
O6—H6*WB*⋯O8^iv^	0.82 (3)	2.02 (3)	2.837 (3)	179 (4)
O7—H7*WA*⋯O1^iii^	0.88 (3)	1.91 (3)	2.780 (3)	169 (3)
O7—H7*WB*⋯O8	0.82 (3)	1.90 (3)	2.710 (3)	169 (3)
O8—H8*WA*⋯O9^v^	0.84 (2)	1.93 (2)	2.767 (3)	175 (3)
O8—H8*WB*⋯O4^vi^	0.87 (2)	1.91 (2)	2.755 (3)	164 (3)
O9—H9*WA*⋯O2	0.85 (2)	2.05 (2)	2.830 (3)	152 (3)
O9—H9*WB*⋯O4^ii^	0.83 (2)	2.03 (2)	2.862 (3)	174 (3)

Symmetry codes: (i) 
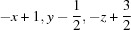
; (ii) 
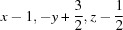
; (iii) 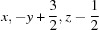; (iv) 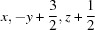; (v) 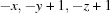; (vi) 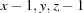.

**Fig. 1.**
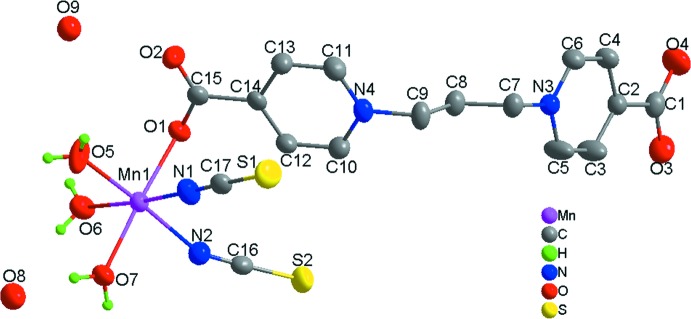
The structure of the title compound with the atom numbering scheme. Displacement ellipsoids are drawn at the 50% probability level. Hydrogen atoms are omitted.

**Fig. 2.**
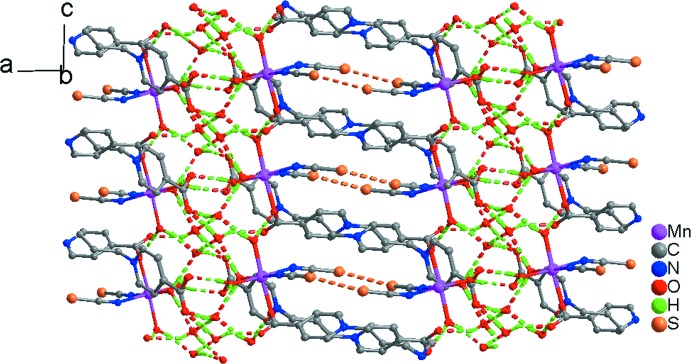
View of the intermolecular interactions in the title compound. Hydrogen bonds and S···S contacts are drawn with dashed lines. Hydrogen atoms attached to C atoms are omitted.

## supplementary crystallographic information

### 1. Comment

The double betaine 1,3-bis(pyridinium-4-carboxylato)propane is a neutral dicarboxylate ligand. In recent years, an increasing number of coordination compounds with this ligand have been reported (Jiang *et al.*, 2006; Li *et al.*, 2007; Wu *et al.*, 2006; Zhang *et al.*, 2002). The structure of the title compound consists of the mononuclear complex molecules [Mn(C_15_O_4_N_2_H_14_)(NCS)_2_(H_2_O)_3_] and lattice water molecules. The metal ion adopts an octahedral coordination geometry completed by three water molecules (O5, O6 and O7), a carboxylato O atom (O1) from the betaine ligand, and two N atoms (N1 and N2) from two thiocyanato anions in *cis* position (Fig. 1). There is a lot of intermolecular O—H···O hydrogen bonds involving the carboxylate groups and water molecules. The O5 water molecule forms two hydrogen bonds with uncoordinated carboxylate oxygen atoms (O3) from different complex molecules, the O6 water molecule contributes its hydrogen atoms to two lattice water molecules (O8 and O9), and the O6 water molecule forms two hydrogen bonds with the coordinated O1 atom and a lattice water molecule (O8). The lattice water molecules are four-coordinated: they accept hydrogen atoms from coordinated water molecules and contribute their hydrogen atoms to carboxylate oxygen atoms (O4 and O2) or water molecules. These hydrogen bonds lead to a three-dimensional architecture (Fig. 2). In addition, there is a short S···S contact [S1···S2(1 - *x*, 1 - *y*, 1 - *z*), 3.625 (1) Å] between the thiocyanato ions from neighboring complex molecules.

### 2. Experimental

The preparation of the compound: 1,3-bis(pyridinium-4-carboxylato)propane (0.029 g, 0.10 mmol) and Potassium thiocyanate (0.039 g, 0.40 mmol) dissolved in ethanol (3 ml) and water (5 ml) were added into the ethanol solution of manganese(II) dichloride hexahydrate (0.040 g, 0.2 mmol),and the resulting light yellow solution was stirred for several minutes, filtered. The filtrate was kept at room temprature, and a few days later,light yellow single crystals suitable for X-ray diffraction was obtained.

### 3. Refinement

All hydrogen atoms attached to carbon atoms were placed geometrically at calculated positions and refined using the riding model with secondary C—H = 0.97 Å and aromatic C—H = 0.93 Å and with *U*_iso_(H) = 1.2*U*_eq_(C). H atoms bonded to O atoms have been refined isotropically with *U*_iso_(H) = 1.5*U*_eq_(O). In the two non-coordinating water molecules the O-H distances were restrained to 0.85 (2)Å and the H···H distances to 1.38 (2)Å.

### Crystal data

**Table:**

[Mn(NCS)_2_(C_15_H_14_N_2_O_4_)(H_2_O)_3_]·2H_2_O	*F*(000) = 1132
*M**_r_* = 547.46	*D*_x_ = 1.527 Mg m^−^^3^
Monoclinic, *P*2_1_/*c*	Mo *K*α radiation, λ = 0.71073 Å
*a* = 17.056 (2) Å	Cell parameters from 2306 reflections
*b* = 11.7514 (16) Å	θ = 2.4–23.7°
*c* = 11.8962 (16) Å	µ = 0.78 mm^−^^1^
β = 92.984 (2)°	*T* = 273 K
*V* = 2381.1 (6) Å^3^	Rectangular, yellow
*Z* = 4	0.10 × 0.08 × 0.05 mm

### Data collection

**Table:**

Bruker SMART CCD area-detector diffractometer	5451 independent reflections
Radiation source: fine-focus sealed tube	3409 reflections with *I* > 2σ(*I*)
Graphite monochromator	*R*_int_ = 0.049
phi and ω scans	θ_max_ = 27.5°, θ_min_ = 2.1°
Absorption correction: multi-scan (*SADABS*; Bruker, 1999)	*h* = −20→22
*T*_min_ = 0.926, *T*_max_ = 0.962	*k* = −9→15
14572 measured reflections	*l* = −12→15

### Refinement

**Table:**

Refinement on *F*^2^	Primary atom site location: structure-invariant direct methods
Least-squares matrix: full	Secondary atom site location: difference Fourier map
*R*[*F*^2^ > 2σ(*F*^2^)] = 0.045	Hydrogen site location: inferred from neighbouring sites
*wR*(*F*^2^) = 0.104	H atoms treated by a mixture of independent and constrained refinement
*S* = 1.04	*w* = 1/[σ^2^(*F*_o_^2^) + (0.0366*P*)^2^] where *P* = (*F*_o_^2^ + 2*F*_c_^2^)/3
5451 reflections	(Δ/σ)_max_ < 0.001
328 parameters	Δρ_max_ = 0.34 e Å^−^^3^
6 restraints	Δρ_min_ = −0.33 e Å^−^^3^

### Special details

**Table:**

Geometry. All e.s.d.'s (except the e.s.d. in the dihedral angle between two l.s. planes) are estimated using the full covariance matrix. The cell e.s.d.'s are taken into account individually in the estimation of e.s.d.'s in distances, angles and torsion angles; correlations between e.s.d.'s in cell parameters are only used when they are defined by crystal symmetry. An approximate (isotropic) treatment of cell e.s.d.'s is used for estimating e.s.d.'s involving l.s. planes.
Refinement. Refinement of *F*^2^ against ALL reflections. The weighted *R*-factor *wR* and goodness of fit *S* are based on *F*^2^, conventional *R*-factors *R* are based on *F*, with *F* set to zero for negative *F*^2^. The threshold expression of *F*^2^ > σ(*F*^2^) is used only for calculating *R*-factors(gt) *etc*. and is not relevant to the choice of reflections for refinement. *R*-factors based on *F*^2^ are statistically about twice as large as those based on *F*, and *R*- factors based on ALL data will be even larger.

### Fractional atomic coordinates and isotropic or equivalent isotropic displacement parameters (Å^2^)

**Table:**

	*x*	*y*	*z*	*U*_iso_*/*U*_eq_	
Mn1	0.19350 (2)	0.66835 (4)	0.46440 (3)	0.02899 (13)	
C1	0.91446 (16)	0.7847 (3)	1.0248 (3)	0.0356 (7)	
C2	0.85603 (15)	0.6997 (2)	0.9725 (2)	0.0307 (6)	
C3	0.83380 (19)	0.7061 (3)	0.8598 (3)	0.0515 (9)	
H3A	0.8537	0.7634	0.8155	0.062*	
C4	0.82470 (16)	0.6147 (2)	1.0353 (2)	0.0344 (7)	
H4A	0.8381	0.6094	1.1119	0.041*	
C5	0.78248 (19)	0.6283 (3)	0.8131 (3)	0.0547 (10)	
H5A	0.7680	0.6327	0.7368	0.066*	
C6	0.77363 (15)	0.5376 (2)	0.9852 (2)	0.0348 (7)	
H6A	0.7534	0.4792	1.0278	0.042*	
C7	0.69666 (14)	0.4628 (2)	0.8224 (2)	0.0332 (7)	
H7B	0.6962	0.3943	0.8678	0.040*	
H7A	0.7141	0.4423	0.7488	0.040*	
C8	0.61420 (14)	0.5110 (2)	0.8099 (2)	0.0346 (7)	
H8B	0.6153	0.5865	0.7772	0.042*	
H8A	0.5918	0.5165	0.8830	0.042*	
C9	0.56506 (15)	0.4320 (3)	0.7341 (2)	0.0374 (7)	
H9B	0.5863	0.4306	0.6600	0.045*	
H9A	0.5681	0.3554	0.7644	0.045*	
C10	0.46197 (16)	0.5602 (3)	0.6618 (2)	0.0376 (7)	
H10A	0.5003	0.5987	0.6239	0.045*	
C11	0.42645 (16)	0.4099 (3)	0.7759 (2)	0.0374 (7)	
H11A	0.4405	0.3459	0.8183	0.045*	
C12	0.38622 (15)	0.5987 (2)	0.6539 (2)	0.0338 (7)	
H12A	0.3736	0.6639	0.6126	0.041*	
C13	0.34983 (16)	0.4439 (2)	0.7678 (2)	0.0349 (7)	
H13A	0.3119	0.4019	0.8029	0.042*	
C14	0.32846 (15)	0.5407 (2)	0.7073 (2)	0.0278 (6)	
C15	0.24337 (16)	0.5814 (3)	0.6987 (2)	0.0324 (7)	
C16	0.36520 (17)	0.7912 (2)	0.4339 (2)	0.0315 (6)	
C17	0.29664 (16)	0.4229 (3)	0.4638 (2)	0.0319 (6)	
N1	0.25535 (14)	0.5006 (2)	0.45556 (19)	0.0418 (6)	
N2	0.29939 (14)	0.7665 (2)	0.4249 (2)	0.0422 (6)	
N3	0.75267 (12)	0.5455 (2)	0.87586 (18)	0.0312 (5)	
N4	0.48197 (12)	0.4677 (2)	0.72342 (18)	0.0322 (5)	
O1	0.23085 (10)	0.67351 (16)	0.64340 (14)	0.0335 (5)	
O2	0.19488 (11)	0.52474 (19)	0.74602 (17)	0.0492 (6)	
O3	0.93988 (12)	0.85658 (18)	0.95846 (18)	0.0455 (5)	
O4	0.93195 (12)	0.7747 (2)	1.12736 (17)	0.0525 (6)	
O5	0.08954 (12)	0.5776 (2)	0.4957 (2)	0.0585 (7)	
H5WB	0.046 (2)	0.600 (3)	0.480 (3)	0.088*	
H5WA	0.089 (2)	0.503 (4)	0.508 (3)	0.088*	
O6	0.11913 (13)	0.82309 (19)	0.47446 (18)	0.0421 (6)	
H6WB	0.095 (2)	0.846 (3)	0.527 (3)	0.063*	
H6WA	0.095 (2)	0.835 (3)	0.418 (3)	0.063*	
O7	0.16305 (12)	0.67069 (19)	0.28495 (16)	0.0372 (5)	
H7WB	0.1212 (19)	0.651 (3)	0.255 (3)	0.056*	
H7WA	0.1806 (18)	0.727 (3)	0.244 (3)	0.056*	
O8	0.03603 (12)	0.5969 (2)	0.15924 (19)	0.0514 (6)	
H8WB	−0.0005 (17)	0.648 (2)	0.161 (3)	0.077*	
H8WA	0.0157 (18)	0.5357 (17)	0.181 (3)	0.077*	
O9	0.03881 (12)	0.5981 (2)	0.77207 (17)	0.0453 (6)	
H9WB	0.0092 (15)	0.633 (3)	0.726 (2)	0.068*	
H9WA	0.0805 (13)	0.578 (3)	0.741 (2)	0.068*	
S1	0.35705 (5)	0.31522 (7)	0.47422 (7)	0.0519 (2)	
S2	0.45791 (5)	0.82371 (8)	0.45024 (7)	0.0530 (3)	

### Atomic displacement parameters (Å^2^)

**Table:**

	*U*^11^	*U*^22^	*U*^33^	*U*^12^	*U*^13^	*U*^23^
Mn1	0.0255 (2)	0.0327 (3)	0.0285 (2)	0.00140 (18)	−0.00004 (17)	0.00147 (19)
C1	0.0278 (15)	0.0328 (17)	0.0461 (19)	0.0018 (13)	0.0012 (14)	−0.0026 (14)
C2	0.0262 (15)	0.0340 (17)	0.0316 (16)	−0.0023 (12)	−0.0011 (12)	0.0016 (12)
C3	0.058 (2)	0.061 (2)	0.0353 (18)	−0.0301 (18)	−0.0047 (15)	0.0120 (16)
C4	0.0382 (16)	0.0374 (17)	0.0271 (15)	−0.0058 (13)	−0.0035 (12)	0.0000 (13)
C5	0.060 (2)	0.075 (3)	0.0286 (17)	−0.0327 (19)	−0.0078 (15)	0.0132 (17)
C6	0.0377 (16)	0.0349 (18)	0.0318 (16)	−0.0026 (13)	0.0002 (13)	0.0072 (13)
C7	0.0270 (14)	0.0346 (17)	0.0378 (16)	−0.0027 (12)	0.0001 (12)	−0.0048 (13)
C8	0.0278 (15)	0.0332 (17)	0.0427 (17)	−0.0001 (13)	0.0005 (13)	−0.0046 (14)
C9	0.0258 (15)	0.0373 (18)	0.0484 (18)	0.0022 (13)	−0.0039 (13)	−0.0076 (14)
C10	0.0310 (16)	0.0431 (19)	0.0388 (17)	−0.0070 (14)	0.0003 (13)	0.0057 (15)
C11	0.0383 (17)	0.0330 (18)	0.0401 (17)	−0.0016 (14)	−0.0050 (13)	0.0041 (14)
C12	0.0321 (15)	0.0382 (18)	0.0307 (15)	−0.0030 (13)	−0.0013 (12)	0.0089 (13)
C13	0.0309 (15)	0.0367 (18)	0.0371 (17)	−0.0036 (13)	0.0019 (13)	0.0064 (14)
C14	0.0291 (14)	0.0335 (16)	0.0203 (13)	0.0009 (12)	−0.0037 (11)	−0.0013 (12)
C15	0.0331 (15)	0.0409 (19)	0.0232 (15)	0.0017 (13)	0.0007 (12)	−0.0001 (13)
C16	0.0367 (17)	0.0302 (16)	0.0277 (15)	0.0013 (13)	0.0023 (13)	0.0057 (12)
C17	0.0336 (15)	0.0372 (18)	0.0245 (15)	−0.0003 (13)	−0.0006 (12)	−0.0033 (13)
N1	0.0423 (15)	0.0457 (17)	0.0366 (14)	0.0102 (13)	−0.0048 (12)	−0.0057 (12)
N2	0.0329 (14)	0.0525 (18)	0.0411 (15)	−0.0043 (12)	0.0002 (12)	0.0073 (13)
N3	0.0279 (12)	0.0370 (15)	0.0285 (13)	−0.0037 (10)	−0.0008 (10)	−0.0009 (11)
N4	0.0281 (12)	0.0339 (14)	0.0341 (13)	−0.0001 (11)	−0.0037 (10)	−0.0039 (11)
O1	0.0367 (11)	0.0362 (12)	0.0275 (10)	0.0079 (9)	0.0001 (8)	0.0023 (9)
O2	0.0328 (11)	0.0633 (16)	0.0522 (13)	0.0026 (11)	0.0087 (10)	0.0228 (12)
O3	0.0421 (12)	0.0357 (13)	0.0583 (14)	−0.0128 (10)	−0.0005 (10)	0.0030 (11)
O4	0.0566 (14)	0.0599 (16)	0.0394 (13)	−0.0165 (12)	−0.0121 (11)	−0.0053 (11)
O5	0.0266 (11)	0.0391 (14)	0.110 (2)	0.0006 (11)	0.0038 (13)	0.0183 (14)
O6	0.0475 (14)	0.0417 (13)	0.0371 (13)	0.0119 (11)	0.0029 (10)	0.0027 (11)
O7	0.0348 (12)	0.0452 (14)	0.0307 (12)	−0.0068 (10)	−0.0047 (9)	0.0059 (10)
O8	0.0427 (13)	0.0564 (17)	0.0543 (14)	−0.0137 (11)	−0.0044 (11)	0.0116 (13)
O9	0.0388 (13)	0.0530 (15)	0.0442 (13)	0.0006 (11)	0.0019 (10)	0.0046 (11)
S1	0.0514 (5)	0.0444 (5)	0.0606 (6)	0.0178 (4)	0.0094 (4)	0.0025 (4)
S2	0.0334 (4)	0.0735 (7)	0.0513 (5)	−0.0135 (4)	−0.0067 (4)	0.0118 (5)

### Geometric parameters (Å, º)

**Table:**

Mn1—O5	2.118 (2)	C10—N4	1.345 (3)
Mn1—O7	2.171 (2)	C10—C12	1.367 (4)
Mn1—O1	2.1914 (18)	C10—H10A	0.9300
Mn1—N2	2.214 (2)	C11—N4	1.346 (3)
Mn1—O6	2.224 (2)	C11—C13	1.365 (4)
Mn1—N1	2.241 (3)	C11—H11A	0.9300
C1—O4	1.246 (3)	C12—C14	1.379 (3)
C1—O3	1.249 (3)	C12—H12A	0.9300
C1—C2	1.521 (4)	C13—C14	1.385 (4)
C2—C4	1.373 (4)	C13—H13A	0.9300
C2—C3	1.375 (4)	C14—C15	1.526 (4)
C3—C5	1.364 (4)	C15—O2	1.222 (3)
C3—H3A	0.9300	C15—O1	1.278 (3)
C4—C6	1.372 (4)	C16—N2	1.159 (3)
C4—H4A	0.9300	C16—S2	1.628 (3)
C5—N3	1.342 (4)	C17—N1	1.154 (3)
C5—H5A	0.9300	C17—S1	1.632 (3)
C6—N3	1.334 (3)	O5—H5WB	0.81 (4)
C6—H6A	0.9300	O5—H5WA	0.89 (4)
C7—N3	1.482 (3)	O6—H6WB	0.82 (3)
C7—C8	1.516 (3)	O6—H6WA	0.78 (3)
C7—H7B	0.9700	O7—H7WB	0.82 (3)
C7—H7A	0.9700	O7—H7WA	0.88 (3)
C8—C9	1.516 (4)	O8—H8WB	0.867 (17)
C8—H8B	0.9700	O8—H8WA	0.844 (17)
C8—H8A	0.9700	O9—H9WB	0.834 (16)
C9—N4	1.477 (3)	O9—H9WA	0.854 (16)
C9—H9B	0.9700	S1—S2^i^	3.6247 (12)
C9—H9A	0.9700		
			
O5—Mn1—O7	91.13 (9)	N4—C9—H9B	109.2
O5—Mn1—O1	92.68 (9)	C8—C9—H9B	109.2
O7—Mn1—O1	176.17 (8)	N4—C9—H9A	109.2
O5—Mn1—N2	177.44 (9)	C8—C9—H9A	109.2
O7—Mn1—N2	86.61 (8)	H9B—C9—H9A	107.9
O1—Mn1—N2	89.57 (8)	N4—C10—C12	121.1 (3)
O5—Mn1—O6	85.24 (9)	N4—C10—H10A	119.5
O7—Mn1—O6	86.25 (8)	C12—C10—H10A	119.5
O1—Mn1—O6	93.60 (7)	N4—C11—C13	120.9 (3)
N2—Mn1—O6	93.40 (9)	N4—C11—H11A	119.6
O5—Mn1—N1	88.05 (9)	C13—C11—H11A	119.6
O7—Mn1—N1	93.10 (8)	C10—C12—C14	120.0 (3)
O1—Mn1—N1	87.49 (8)	C10—C12—H12A	120.0
N2—Mn1—N1	93.28 (9)	C14—C12—H12A	120.0
O6—Mn1—N1	173.24 (9)	C11—C13—C14	120.2 (3)
O4—C1—O3	127.5 (3)	C11—C13—H13A	119.9
O4—C1—C2	117.2 (3)	C14—C13—H13A	119.9
O3—C1—C2	115.3 (3)	C12—C14—C13	118.0 (3)
C4—C2—C3	118.4 (3)	C12—C14—C15	120.9 (2)
C4—C2—C1	121.3 (3)	C13—C14—C15	121.1 (2)
C3—C2—C1	120.3 (3)	O2—C15—O1	126.8 (3)
C5—C3—C2	119.9 (3)	O2—C15—C14	117.5 (3)
C5—C3—H3A	120.0	O1—C15—C14	115.6 (2)
C2—C3—H3A	120.0	N2—C16—S2	178.2 (3)
C6—C4—C2	120.0 (3)	N1—C17—S1	178.4 (3)
C6—C4—H4A	120.0	C17—N1—Mn1	168.1 (2)
C2—C4—H4A	120.0	C16—N2—Mn1	155.2 (2)
N3—C5—C3	120.9 (3)	C6—N3—C5	120.2 (2)
N3—C5—H5A	119.6	C6—N3—C7	120.4 (2)
C3—C5—H5A	119.6	C5—N3—C7	119.4 (2)
N3—C6—C4	120.6 (3)	C10—N4—C11	119.8 (2)
N3—C6—H6A	119.7	C10—N4—C9	119.4 (2)
C4—C6—H6A	119.7	C11—N4—C9	120.8 (2)
N3—C7—C8	111.8 (2)	C15—O1—Mn1	120.58 (17)
N3—C7—H7B	109.3	Mn1—O5—H5WB	125 (3)
C8—C7—H7B	109.3	Mn1—O5—H5WA	123 (2)
N3—C7—H7A	109.3	H5WB—O5—H5WA	110 (3)
C8—C7—H7A	109.3	Mn1—O6—H6WB	128 (2)
H7B—C7—H7A	107.9	Mn1—O6—H6WA	113 (3)
C7—C8—C9	108.1 (2)	H6WB—O6—H6WA	109 (4)
C7—C8—H8B	110.1	Mn1—O7—H7WB	126 (2)
C9—C8—H8B	110.1	Mn1—O7—H7WA	119 (2)
C7—C8—H8A	110.1	H7WB—O7—H7WA	107 (3)
C9—C8—H8A	110.1	H8WB—O8—H8WA	106 (2)
H8B—C8—H8A	108.4	H9WB—O9—H9WA	110 (2)
N4—C9—C8	112.0 (2)	C17—S1—S2^i^	154.96 (11)

Symmetry code: (i) −*x*+1, −*y*+1, −*z*+1.

### Hydrogen-bond geometry (Å, º)

**Table:**

*D*—H···*A*	*D*—H	H···*A*	*D*···*A*	*D*—H···*A*
O5—H5*WA*···O3^ii^	0.89 (4)	1.84 (4)	2.707 (3)	164 (4)
O5—H5*WB*···O3^iii^	0.81 (4)	1.88 (4)	2.682 (3)	174 (4)
O6—H6*WA*···O9^iv^	0.78 (3)	2.09 (3)	2.862 (3)	168 (4)
O6—H6*WB*···O8^v^	0.82 (3)	2.02 (3)	2.837 (3)	179 (4)
O7—H7*WA*···O1^iv^	0.88 (3)	1.91 (3)	2.780 (3)	169 (3)
O7—H7*WB*···O8	0.82 (3)	1.90 (3)	2.710 (3)	169 (3)
O8—H8*WA*···O9^vi^	0.84 (2)	1.93 (2)	2.767 (3)	175 (3)
O8—H8*WB*···O4^vii^	0.87 (2)	1.91 (2)	2.755 (3)	164 (3)
O9—H9*WA*···O2	0.85 (2)	2.05 (2)	2.830 (3)	152 (3)
O9—H9*WB*···O4^iii^	0.83 (2)	2.03 (2)	2.862 (3)	174 (3)

Symmetry codes: (ii) −*x*+1, *y*−1/2, −*z*+3/2; (iii) *x*−1, −*y*+3/2, *z*−1/2; (iv) *x*, −*y*+3/2, *z*−1/2; (v) *x*, −*y*+3/2, *z*+1/2; (vi) −*x*, −*y*+1, −*z*+1; (vii) *x*−1, *y*, *z*−1.
